# Prognostic and biological significance of MRPL13 in multiple myeloma: evidence from multi-cohort, single-cell analyses

**DOI:** 10.3389/fonc.2026.1880529

**Published:** 2026-08-05

**Authors:** Wanyi Liu, Changjian Yan, Huiqiang Wu, Chaoling Wu, Zhiyin Cai, Zechuan Wang, Weihuang Zhuang, Meiling Li, Jianxin Guo

**Affiliations:** 1 Department of Hematology, The Second Affiliated Hospital of Fujian Medical University, Quanzhou, Fujian, China; 2 The Second Clinical Medical College, Fujian Medical University, Fujian, China; 3 Department of Respiratory Medicine, Affiliated Hospital of Jiujiang University, Jiujiang, China

**Keywords:** biomarker, cell cycle, mitoribosome, MRPL13, multiple myeloma, prognosis

## Abstract

**Background:**

Multiple myeloma (MM) is a clonal plasma cell malignancy with high heterogeneity and poor prognosis. Mitochondrial ribosomal protein L13 (MRPL13) has been linked to tumor progression, but its role in MM remains unclear.

**Methods:**

We analyzed seven datasets comprising 3,117 patient samples from the Multiple Myeloma Research Foundation (MMRF) CoMMpass study (referred to as the TCGA_MMRF cohort) and the Gene Expression Omnibus (GEO) database to evaluate the association between MRPL13 expression and overall survival (OS). Event-free survival (EFS) analysis was performed in the GSE24080 cohort, and progression-free survival (PFS) analysis was conducted in the GSE136337 cohort. We further analyzed single-cell transcriptomic profiles of 74,986 cells from 24 MM patients to delineate the expression landscape of MRPL13 across distinct cellular populations and functional subclusters and to assess its relationship with the immune microenvironment. We performed functional assays involving siRNA-mediated MRPL13 silencing to investigate its effects on MM cell proliferation and cell-cycle progression *in vitro*.

**Results:**

High MRPL13 expression was associated with adverse clinical features and poor survival and was identified as a potential independent prognostic factor. Single-cell analysis revealed enrichment in metabolically active malignant plasma cells and an immunosuppressive microenvironment. Silencing MRPL13 inhibited proliferation and induced G0/G1 arrest.

**Conclusion:**

MRPL13 predicts poor prognosis and promotes MM progression through metabolic and immune regulation, supporting its potential as a prognostic biomarker and therapeutic target.

## 1 Introduction

Multiple myeloma (MM) is a clonal plasma cell malignancy arising in the bone marrow, accounting for approximately 10% of all hematologic cancers (1). Its hallmark features include uncontrolled proliferation of malignant plasma cells, excessive secretion of monoclonal immunoglobulins, and multi-organ involvement (2). Over the past few decades, the advent of novel therapeutic agents, such as proteasome inhibitors, immunomodulatory agents, and monoclonal antibodies, has markedly improved patient outcomes, leading to a substantial extension of median overall survival (3, 4). Nevertheless, MM remains incurable in the majority of patients, with relapse and drug resistance occurring almost inevitably during the disease course. Thus, the identification of novel molecular biomarkers capable of predicting disease progression, refining prognostication, and guiding personalized therapeutic strategies remains an urgent clinical priority.

Mitochondrial function plays a central role in cancer biology, orchestrating metabolic reprogramming, sustaining cell proliferation, and facilitating immune evasion (5). Mitochondrial ribosomal proteins (MRPs), encoded by nuclear genes, translocate into mitochondria, where they assemble with rRNA to form mitoribosomes, thereby regulating mitochondrial protein synthesis and energy metabolism (6). Among these, mitochondrial ribosomal protein L13 (MRPL13) has been reported to be upregulated across multiple solid tumors and is strongly associated with enhanced proliferation, migratory potential, and unfavorable prognosis (7, 8). However, its role in the pathogenesis of MM and its potential influence on the immune microenvironment remain largely unexplored.

In this study, we conducted an integrated analysis of multi-cohort transcriptomic and clinical datasets, combined with single-cell transcriptomics and *in vitro* functional assays, to comprehensively characterize MRPL13 expression in MM and evaluate its prognostic significance. Our findings aim to elucidate the potential of MRPL13 as both a prognostic biomarker and a therapeutic target in MM.

## 2 Materials and methods

### 2.1 Data sources

The transcriptomic and clinical data analyzed in this study were obtained from the Gene Expression Omnibus (GEO) database (https://www.ncbi.nlm.nih.gov/gds/) and The Cancer Genome Atlas (TCGA) database (https://cancergenome.nih.gov/), comprising a total of 3,117 patients with multiple myeloma (MM). Specifically, 843 cases were derived from the TCGA_MMRF cohort, and 2,274 cases from GEO datasets. The datasets included the following: TCGA_MMRF (n = 843) (9), GSE24080 (n = 559; bone marrow CD138^+^plasma cells) (10), GSE2658 (n = 559; bone marrow CD138^+^plasma cells) (11), GSE9782 (n = 264; bone marrow CD138^+^ plasma cells) (12), GSE57317 (n = 55; bone marrow CD138^+^plasma cells) (13), GSE4581 (n = 411; bone marrow CD138^+^plasma cells) (14), and GSE136337 (n = 426; post-treatment patients, bone marrow CD138^+^plasma cells) (15). Detailed clinical characteristics of the included cohorts are summarized in **Supplementary Table 1**.

To minimize potential batch effects arising from different sequencing and microarray platforms, each dataset was analyzed independently using its original platform-specific normalization procedures, without merging raw expression matrices across cohorts. This cohort-specific analytical strategy ensured that all statistical analyses were performed within homogeneous datasets, thereby avoiding cross-platform biases and preserving data comparability.

### 2.2 Gene expression and patient stratification

The survminer R package was used to determine the optimal cutoff value of MRPL13 expression within each dataset based on maximally selected rank statistics (the Maxstat method implemented in the surv_cutpoint function). Patients were subsequently stratified into high-expression and low-expression groups according to the cohort-specific cutoff values.

The optimal cutoff values were independently calculated for each cohort to ensure robustness and avoid arbitrary grouping. The exact cutoff values were as follows: GSE2658 (9.91), GSE24080 (9.97), GSE9782 (6.62), GSE136337 (6.757905), GSE57317 (10.00), GSE4581 (11.27), and TCGA_MMRF (9.807355) (**Supplementary Table 2**).

The association between MRPL13 expression and survival outcomes was first evaluated in the training cohort, GSE24080, and subsequently validated across independent cohorts, including GSE2658, GSE9782, GSE57317, GSE4581, GSE136337, and TCGA_MMRF.

Survival endpoints were defined as follows. Overall survival (OS) was defined as the time from diagnosis to death from any cause. Progression-free survival (PFS) was defined as the time from diagnosis to the first documented disease progression or death, whichever occurred first. Event-free survival (EFS) was defined as the time from diagnosis to the occurrence of any event, including disease progression, treatment discontinuation, or death.

### 2.3 Clinical correlation and molecular feature analysis

Baseline clinical characteristics were compared between the high- and low-MRPL13-expression groups within the GSE24080 and GSE136337 cohorts. Variables assessed included sex, age, ISS stage, R-ISS stage, biochemical parameters, cytogenetic abnormalities, 1q21 amplification, and GEP70 (70-gene expression profiling risk model) molecular subtype. Categorical variables were analyzed using the χ² test or Fisher’s exact test, while continuous variables were compared using the Student’s t-test or one-way analysis of variance (ANOVA). The relationship between 1q21 amplification copy number and MRPL13 expression was further assessed using ANOVA followed by post-hoc multiple-comparison testing.

### 2.4 Single-cell data collection and processing

Single-cell RNA sequencing (scRNA-seq) data were obtained from bone marrow samples of 24 patients with multiple myeloma (https://www.nature.com/articles/s41467-021-22804-x). For each sample, the proportion of mitochondrial gene expression was calculated, and cells were filtered according to the following quality-control criteria: mitochondrial gene percentage <15%, total UMI counts >1,000, and the number of detected genes >500. Doublets were identified and removed using the Scrublet algorithm (default parameters), and batch effects were corrected with the Harmony algorithm. After quality control, a total of 74,986 high-quality single cells were retained. Cells were clustered using standard workflows and annotated based on canonical lineage-defining marker genes. All preprocessing and clustering analyses were performed using the Scanpy software package.

#### 2.4.1 Differential expression analysis

Differentially expressed genes (DEGs) between distinct cell types or subgroups were identified using the Presto R package. Genes with |log2 fold change| > 1 and an adjusted p-value < 0.05 (Benjamini–Hochberg false discovery rate correction) were considered significantly differentially expressed. Results were visualized using the ggplot2 package (R software v4.2.3).

#### 2.4.2 Gene set enrichment analysis

Functional enrichment analysis was performed using the GSEA R package. Gene Ontology Biological Process (GOBP) gene sets were obtained from the Molecular Signatures Database (MSigDB). Pathways with a false discovery rate (FDR) < 0.05 were considered significantly enriched and visualized using R-based plotting tools.

#### 2.4.3 Cell–cell communication analysis

To investigate intercellular interactions in untreated, newly diagnosed MM patients, particularly between tumor cells with high MRPL13 expression and other cell populations, the CellChat R package was applied. A CellChat object was constructed using the createCellChat function, followed by computation of intercellular communication probabilities using computeCommunProb and pathway-level signaling using computeCommunProbPathway. Visualization of communication networks was performed using the built-in CellChat functions.

All preprocessing and clustering analyses were performed using Scanpy software (v1.9.3).

### 2.5 Cell culture and reagents

KM3 and RPMI-8226 multiple myeloma cell lines were kindly provided by Dr. Wenqing Long (Zhengzhou University, China) and originally obtained from the Cell Bank of the Chinese Academy of Sciences (Shanghai, China). Cells were cultured in RPMI-1640 medium supplemented with 10% fetal bovine serum (FBS; Basal Media, China) under standard conditions (37 °C, 5% CO_2_).

Reagents included TRIzol (Vazyme, China), the GoScript™ reverse transcription kit (Promega, USA), SYBR Green qPCR Master Mix (Yeasen, China), antibodies against MRPL13 and β-actin (Proteintech, China), and the CCK-8 and ECL detection kits (ABK BIO, China). Other routine laboratory chemicals were obtained from commercial sources.

### 2.6 siRNA transfection

siRNAs specifically targeting MRPL13 (Magen Biotech, China) were transfected into KM3 and RPMI-8226 cells using Lipofectamine 2000 (Thermo Fisher Scientific, USA) according to the manufacturer’s instructions. The final siRNA concentration was 50 nM. Two siRNA sequences were used: h-MRPL13-siRNA#1: 5′-TGAGGGATCCAGTGGCAATTG-3′ and h-MRPL13-siRNA#2: 5′-CCACCTGAAGATTATCGGCTA-3′.

### 2.7 Quantitative reverse‐transcription polymerase chain reaction

Total RNA was extracted using TRIzol reagent, and cDNA was synthesized using a commercial reverse transcription kit. Relative mRNA expression of MRPL13 was quantified by qRT-PCR (Applied Biosystems 7500, Thermo Fisher Scientific, USA) using GAPDH as the internal control. Data analysis was performed using the 2^-^ΔΔCt method, as previously described (16). Primer sequences were as follows: MRPL13 forward: 5′-ACATAAACCTGTGTTACCATGCAC-3′;MRPL13 reverse: 5′-GGTAGCCAGTATGCGAAGAGT-3′; GAPDH forward: 5′-ACCACAGTCCATGCCATCAC-3′; and GAPDH reverse: 5′-TCCACCACCCTGTTGCTGTA-3′.

### 2.8 Cell counting kit-8 proliferation assay

Cell proliferation was evaluated using the CCK-8 assay (ABK BIO, China) according to the manufacturer’s instructions. Briefly, KM3 and RPMI-8226 cells were seeded into 96-well plates at a density of 5 × 10³ cells/well, incubated for 24–96 h, and absorbance was measured at 450 nm.

### 2.9 Western blotting

Protein extraction, separation, and transfer were carried out using standard protocols (17). Membranes were probed with antibodies against MRPL13 (1:2000, Proteintech, China) and β-actin (1:20000, Proteintech, China), followed by an HRP-conjugated secondary antibody (Tagene Bio, China). Protein bands were visualized by enhanced chemiluminescence (ABK BIO, China).

### 2.10 Flow cytometry analysis

#### 2.10.1 Apoptosis assay

Transfected KM3 and RPMI-8226 cells were collected and stained using an Annexin V-APC/7-AAD apoptosis detection kit according to the manufacturer’s instructions. After staining, samples were immediately analyzed using a BD FACS Canto II flow cytometer (BD Biosciences).

#### 2.10.2 Cell cycle analysis

To evaluate cell cycle distribution, transfected cells were fixed in ice-cold 75% ethanol at −20 °C overnight. After fixation, cells were washed with PBS and stained with propidium iodide (PI) solution containing RNase A. DNA content was then analyzed using a BD FACS Canto II flow cytometer.

Flow cytometry data were analyzed using FlowJo software (v10.8.1) using standard gating strategies.

### 2.11 Statistical analysis

All analyses were performed using R (v4.2.3) and GraphPad Prism 9.0. For normally distributed data, Student’s t-test (for two groups) or one-way ANOVA (for multiple groups) was used; non-parametric data were analyzed using the Mann–Whitney U test or the Kruskal–Wallis test. Kaplan–Meier survival analysis and Cox proportional hazards regression were conducted for prognostic evaluation. P < 0.05 was considered statistically significant.

Data visualization was performed using R software. Specifically, volcano plots and general statistical figures were generated using ggplot2, heatmaps were created using the pheatmap package, and Kaplan–Meier survival curves were generated using the survminer package. UMAP plots and single-cell visualization were generated using Scanpy-based workflows (Python v3.x, Scanpy v1.9.x), while intercellular communication networks were visualized using the CellChat package.

## 3 Results

### 3.1 Baseline clinical characteristics of 3,117 patients with multiple myeloma

**Figure 1:**
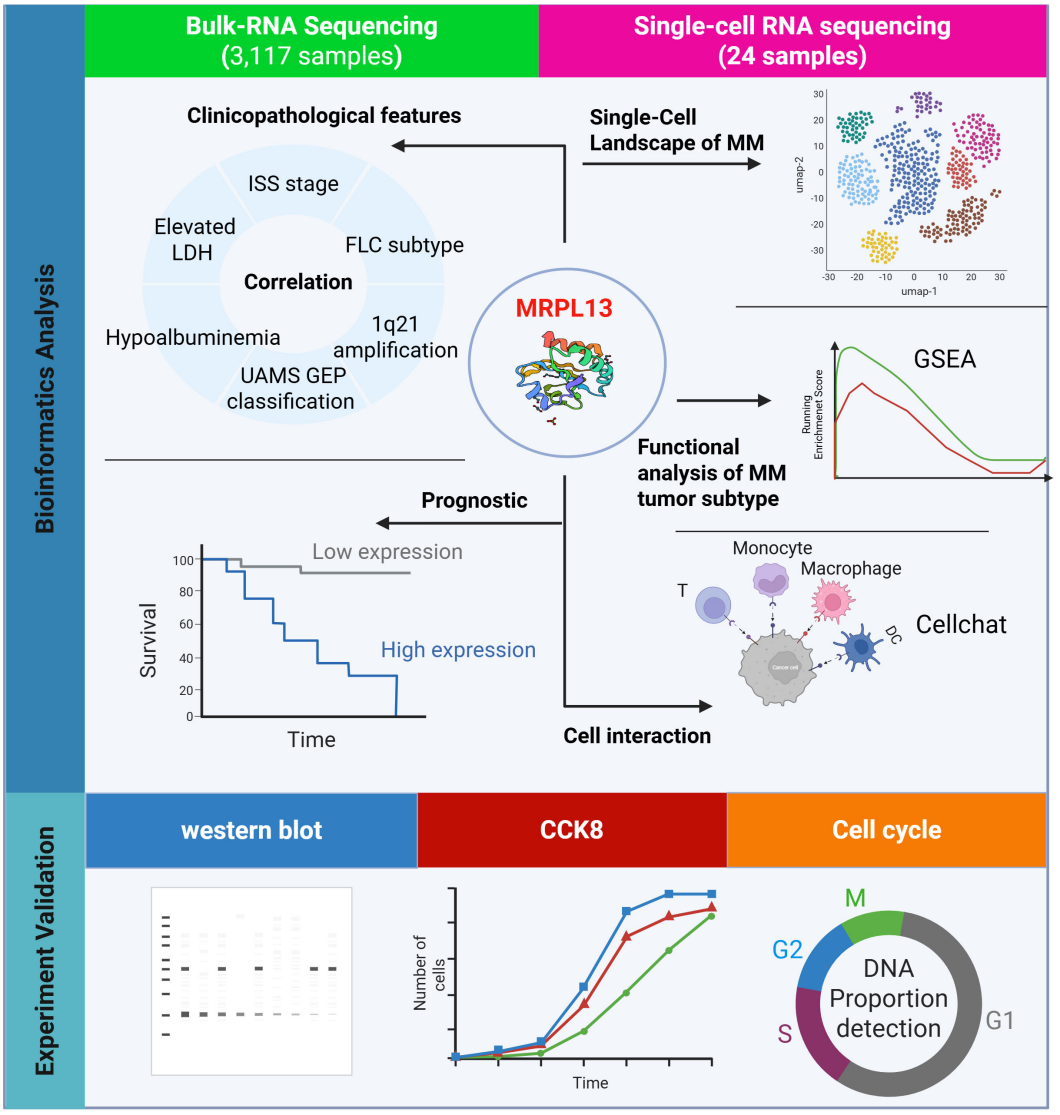
Schematic overview of the study design and analytical workflow. Bulk RNA-sequencing (n = 3,117 samples) was used to evaluate clinicopathological correlations and prognostic implications of MRPL13, including ISS stage, LDH, albumin, FLC subtype, and 1q21 amplification. Single-cell RNA sequencing (24 samples) delineated the cellular landscape of multiple myeloma (MM), functional heterogeneity of plasma/tumor cell subclusters, and immune cell interactions using GSEA and CellChat analyses. Experimental validation included CCK-8 proliferation assays and flow cytometric cell cycle detection, confirming the biological role of MRPL13 in promoting MM progression.

This study included a total of 3,117 patients with multiple myeloma (MM) from six GEO datasets and one TCGA_MMRF dataset (**Figure 1**; **Supplementary Table 1**). Among them, GSE24080 (n = 559) served as the training cohort, while the remaining datasets (GSE2658, GSE9782, GSE136337, GSE57317, GSE4581, and TCGA_MMRF) were used as validation cohorts. All samples were profiled across four platforms (GPL27143, GPL570, GPL96, and TCGA_MMRF), with GPL570 being the most frequently used, accounting for 69.66% of cases.

Regarding demographic characteristics, 59.70% of the patients were men, and 40.30% were women. The age distribution indicated that 52.20% of patients were <60 years old, while 47.80% were ≥60 years old. The racial composition was predominantly White/Caucasian (89.23%), followed by Black (9.15%), Asian (1.06%), Hispanic (0.37%), and Native American (0.19%).

With respect to disease stage, ISS stage I, II, and III accounted for 47.15%, 28.41%, and 24.44% of patients, respectively. Information on R-ISS stage was available only for the GSE136337 cohort, in which 64.44% of patients were classified as R-ISS stage II.

### 3.2 High MRPL13 expression predicts poor prognosis in multiple myeloma

**Figure 2:**
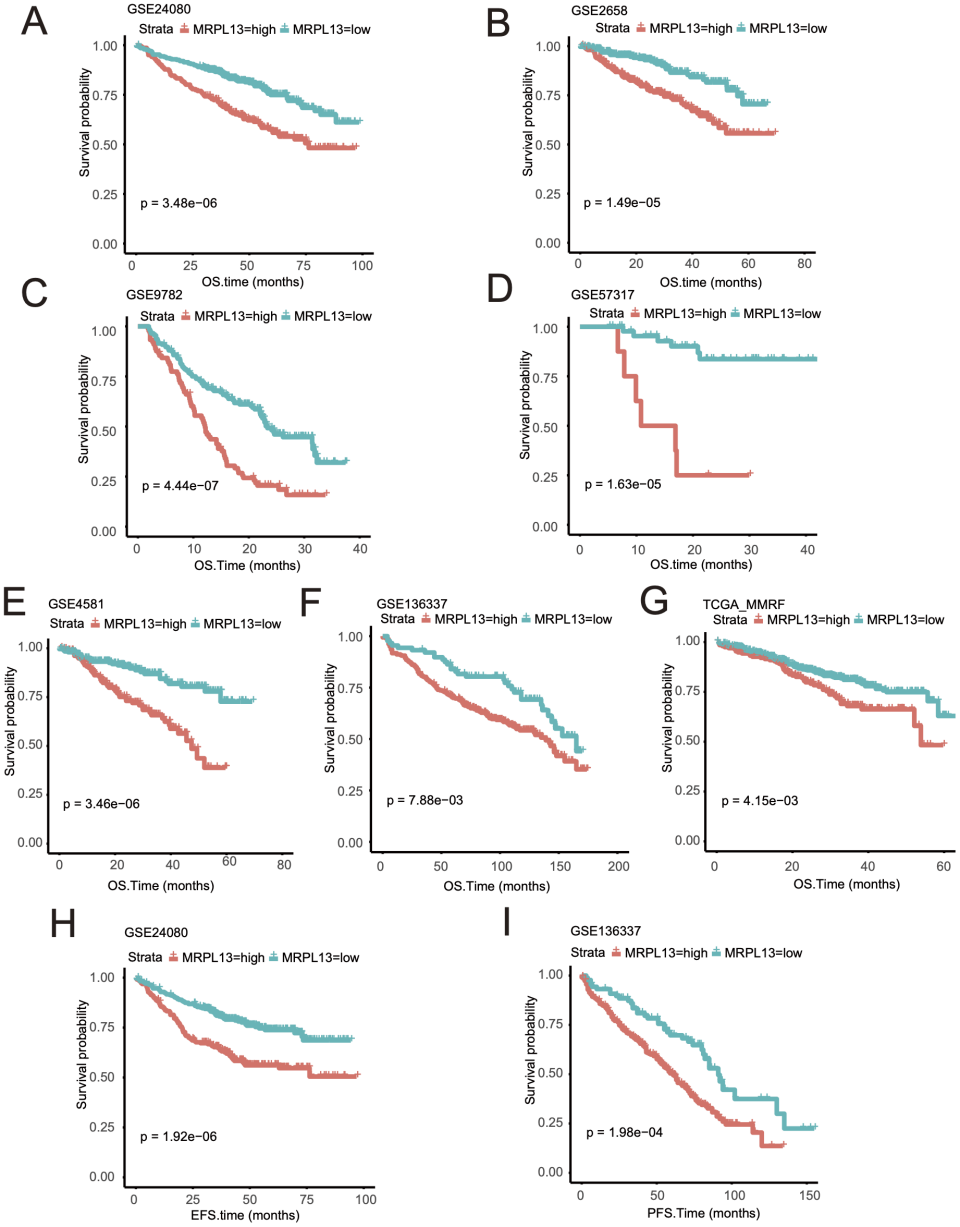
Prognostic impact of MRPL13 expression in multiple myeloma. **(A–I)** Kaplan–Meier survival analyses across independent MM cohorts stratified by MRPL13 expression. High MRPL13 expression was consistently associated with inferior overall survival (OS), underscoring its potential as a prognostic biomarker.

In the training cohort, GSE24080, Kaplan–Meier survival analysis demonstrated that patients with high MRPL13 expression had significantly shorter overall survival (OS) compared with those in the low-expression group (p = 3.48e-06; **Figure 2A**). This adverse prognostic trend was consistently validated across multiple independent cohorts, including GSE2658 (p = 1.49e-05; **Figure 2B**), GSE9782 (p = 4.44e-07; **Figure 2C**), GSE57317 (p = 1.63e-05; **Figure 2D**), GSE4581 (p = 3.46e-06; **Figure 2E**), GSE136337 (p = 7.88e-03; **Figure 2F**), and TCGA_MMRF (p = 4.15e-03; **Figure 2G**), where high MRPL13 expression was consistently associated with reduced OS.

Beyond OS, high MRPL13 expression was also strongly correlated with unfavorable event-free survival (EFS) and progression-free survival (PFS). In GSE24080, patients in the high-expression group had significantly shorter EFS compared with those in the low-expression group (p = 1.92e-06; **Figure 2H**). Similarly, in GSE136337, high MRPL13 expression was associated with markedly shorter PFS (p = 1.98e-04; **Figure 2I**).

Collectively, these findings demonstrate that high MRPL13 expression is consistently associated with shortened OS, EFS, and PFS across independent cohorts, supporting its role as a potential biomarker of poor prognosis in MM.

### 3.3 High MRPL13 expression is associated with adverse clinicopathological features

To further investigate the relationship between MRPL13 expression and clinical characteristics, we compared baseline features between the high- and low-expression groups in the GSE24080 cohort (n = 559) (**Table 1**). The high-expression group had a significantly higher proportion of female patients (47.96% vs. 34.32%, p = 0.0017), a greater proportion of ISS stage III patients (25.00% vs. 18.93%), and a lower proportion of ISS stage I patients (46.36% vs. 57.10%, p = 0.0422). With respect to molecular subtypes, the Proliferation (PR) subtype was more frequent in the high-expression group (14.55% vs. 4.50%), whereas the hyperdiploid (HY) subtype was significantly less frequent (12.73% vs. 26.13%, p < 0.0001). Additionally, patients in the high-expression group were more likely to present with Free Light Chain (FLC) subtype disease (19.91% vs. 12.69%, p = 0.0294), elevated LDH (60.18% vs. 43.49%, p = 0.0002), hypoalbuminemia (52.04% vs. 40.24%, p = 0.0079), abnormal MRI findings (57.07% vs. 45.74%, p = 0.0147), cytogenetic abnormalities (43.44% vs. 32.84%, p = 0.0144), and 1q21 amplification (p < 0.0001). Collectively, these findings indicate that high MRPL13 expression is closely associated with multiple adverse clinical characteristics.

**Table 1:** Baseline characteristics of MM Patients in the MRPL13 low and high expression group from GSE24080.

	level	Overall	Low	High	P value
n		559	338	221	
RACE (%)	White	497 (88.91)	306 (90.53)	191 (86.43)	0.1694
	Other	62 (11.09)	32 (9.47)	30 (13.57)	
Age (%)	<60	318 (56.89)	186 (55.03)	132 (59.73)	0.3127
	≥60	241 (43.11)	152 (44.97)	89 (40.27)	
ISS (%)	I	295 (52.87)	193 (57.10)	102 (46.36)	0.0422
	II	144 (25.81)	81 (23.96)	63 (28.64)	
	III	119 (21.33)	64 (18.93)	55 (25.00)	
gender (%)	Female	222 (39.71)	116 (34.32)	106 (47.96)	0.0017
	Male	337 (60.29)	222 (65.68)	115 (52.04)	
Subgroup (%)	CD1	28 (5.06)	11 (3.30)	17 (7.73)	<0.0001
	CD2	58 (10.49)	40 (12.01)	18 (8.18)	
	HY	115 (20.80)	87 (26.13)	28 (12.73)	
	LB	58 (10.49)	38 (11.41)	20 (9.09)	
	MF	36 (6.51)	19 (5.71)	17 (7.73)	
	MS	66 (11.93)	28 (8.41)	38 (17.27)	
	MY	145 (26.22)	95 (28.53)	50 (22.73)	
	PR	47 (8.50)	15 (4.50)	32 (14.55)	
ISOTYPE (%)	FLC	84 (15.58)	41 (12.69)	43 (19.91)	0.0294
	IgA	133 (24.68)	74 (22.91)	59 (27.31)	
	IgD	3 (0.56)	2 (0.62)	1 (0.46)	
	IgG	313 (58.07)	204 (63.16)	109 (50.46)	
	Nonsecretory	6 (1.11)	2 (0.62)	4 (1.85)	
B2M (%)	Low	277 (49.64)	175 (51.78)	102 (46.36)	0.2449
	High	281 (50.36)	163 (48.22)	118 (53.64)	
CRP (%)	Low	275 (49.55)	173 (51.64)	102 (46.36)	0.2586
	High	280 (50.45)	162 (48.36)	118 (53.64)	
CREAT (%)	Low	246 (44.24)	151 (44.94)	95 (43.18)	0.7482
	High	310 (55.76)	185 (55.06)	125 (56.82)	
LDH (%)	Low	279 (49.91)	191 (56.51)	88 (39.82)	0.0002
	High	280 (50.09)	147 (43.49)	133 (60.18)	
ALB (%)	Low	251 (44.90)	136 (40.24)	115 (52.04)	0.0079
	High	308 (55.10)	202 (59.76)	106 (47.96)	
HGB (%)	Low	279 (49.91)	157 (46.45)	122 (55.20)	0.0527
	High	280 (50.09)	181 (53.55)	99 (44.80)	
ASPC (%)	Low	255 (48.02)	160 (49.54)	95 (45.67)	0.435
	High	276 (51.98)	163 (50.46)	113 (54.33)	
BMPC (%)	Low	263 (48.43)	166 (50.61)	97 (45.12)	0.2441
	High	280 (51.57)	162 (49.39)	118 (54.88)	
MRI (%)	Low	260 (49.81)	172 (54.26)	88 (42.93)	0.0147
	High	262 (50.19)	145 (45.74)	117 (57.07)	
Cyto.Abn (%)	No	352 (62.97)	227 (67.16)	125 (56.56)	0.0144
	Yes	207 (37.03)	111 (32.84)	96 (43.44)	
FISH.1q21.Amplification (%)	Without	132 (23.61)	90 (26.63)	42 (19.00)	<0.0001
	2	68 (12.16)	32 (9.47)	36 (16.29)	
	3	44 (7.87)	14 (4.14)	30 (13.57)	
	4+	315 (56.35)	202 (59.76)	113 (51.13)	

n, number of patients; BM_BLAST, bone marrow blast cell; WBC, peripheral blood WBC; PB_BLAST, peripheral blood blast cell; MUD, HSCT of matched unrelated donor; Haplo, haploidentical HSCT; WT, wild type. Unpaired t test (two sides), was used in two group measurement data. ANOVA test was used in in multi groups’ measurement data. Fisher’s exact test was used in enumeration data.

**Figure 3:**
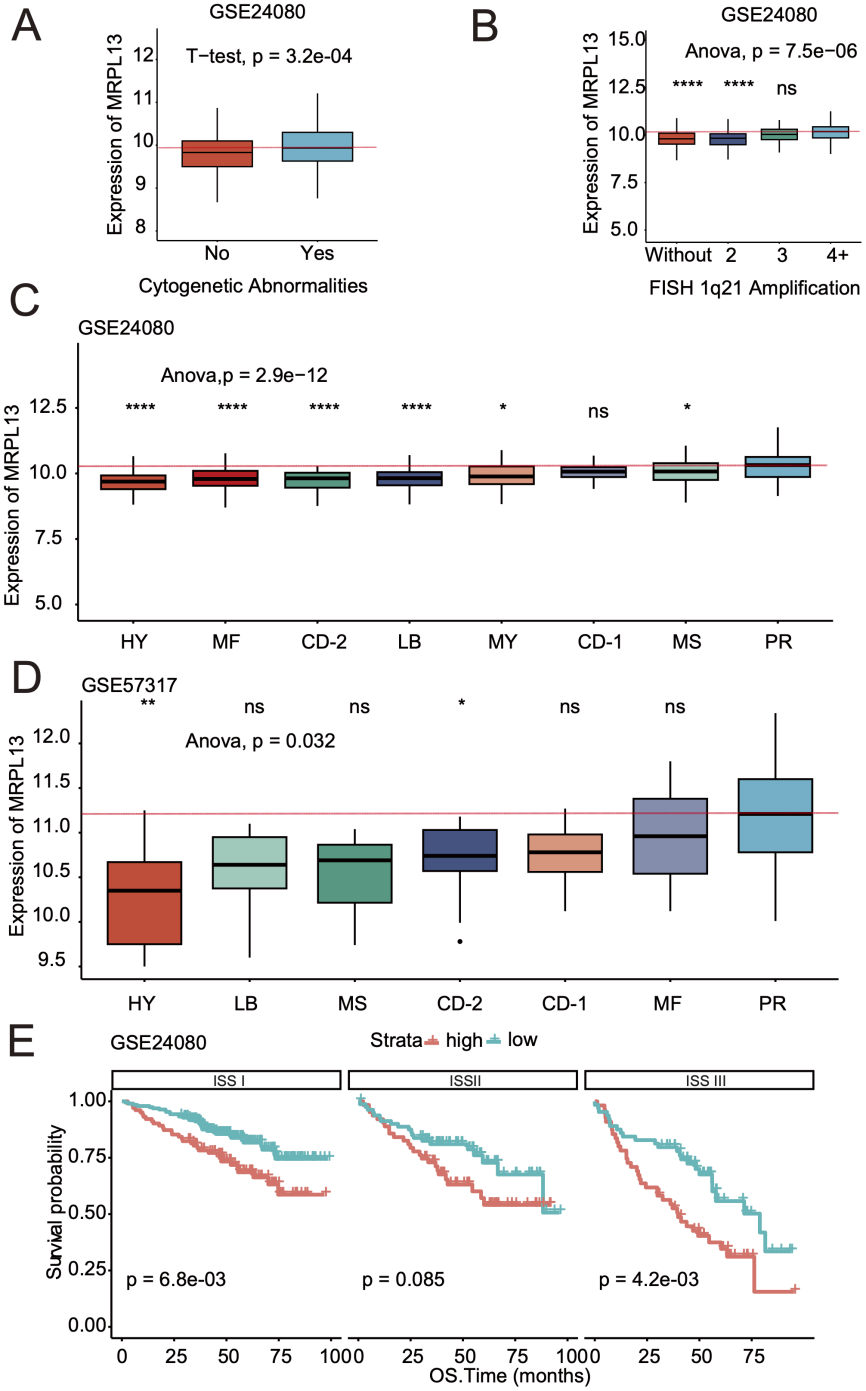
Association of MRPL13 expression with genetic alterations and prognosis in MM. **(A)** Comparison of MRPL13 expression between patients with and without cytogenetic abnormalities in the GSE24080 cohort (ANOVA-based group comparison). **(B)** Association between MRPL13 expression and 1q21 amplification status in the GSE24080 cohort (ANOVA-based comparison across copy number groups). **(C, D)** Differential expression of MRPL13 across molecular subgroups in the GSE24080 and GSE57317 cohorts, respectively (boxplot analysis with group-wise comparisons). **(E)** Kaplan–Meier survival curves illustrating the impact of MRPL13 expression on overall survival (OS) stratified by cytogenetic risk groups. Statistical significance is indicated as follows: ns (not significant), *p < 0.05, **p < 0.01, ***p < 0.001. The red line represents the median expression level of the high-expression group.

Molecular analyses further supported these observations. Patients with cytogenetic abnormalities exhibited significantly higher MRPL13 expression compared with those without abnormalities (**Figure 3A**; Student’s t-test, p = 3.2e-04). ANOVA revealed a stepwise increase in MRPL13 expression with increasing 1q21 copy number (**Figure 3B**; p = 7.5e-06), with significant differences observed between the no-gain group and the 2-copy and 4-copy gain groups (p < 0.001), but not between the no-gain group and the 3-copy group. Regarding molecular subtypes, MRPL13 expression varied significantly among subtypes in the GSE24080 cohort (**Figure 3C**; p = 2.9e-12), with expression highest in the PR subtype and lowest in the HY subtype (both p < 0.0001). This pattern was validated in the GSE57317 cohort (**Figure 3D**; p = 0.032), where MRPL13 expression was significantly higher in the PR subtype than in the HY subtype (p < 0.01).

Stratified survival analyses (**Figure 3E**) further demonstrated that, within the GSE24080 cohort, high MRPL13 expression was associated with significantly shorter OS in both ISS stage I (p = 6.8e-03) and ISS stage III (p = 4.2e-03) subgroups. Although the difference did not reach statistical significance in the ISS stage II subgroup (p = 0.085), the same adverse trend was observed.

### 3.4 High MRPL13 expression is a potential prognostic factor in MM

In the GSE24080 cohort, univariate Cox regression identified multiple clinical variables significantly associated with OS and EFS. For OS, adverse prognostic factors included low albumin (HR = 0.482, p = 2.46e-06), low hemoglobin (HR = 0.662, p = 7.56e-03), higher plasma cell infiltration (abnormal plasma cell percentage [ASPC], high vs. low: HR = 1.88, p = 1.25e-04), elevated β_2_-microglobulin (HR = 2.30, p = 2.38e-07), high LDH (HR = 2.19, p = 1.01e-06), MRI abnormalities (HR = 1.86, p = 1.11e-04), cytogenetic abnormalities (HR = 2.29, p = 6.01e-08), and high MRPL13 expression (HR = 2.01, p = 5.41e-06).

Similarly, univariate analyses of EFS demonstrated consistent associations. Low albumin (HR = 0.599, p = 5.61e-05), high ASPC (HR = 1.60, p = 3.84e-04), elevated β_2_-microglobulin (HR = 2.01, p = 9.67e-08), high LDH (HR = 1.79, p = 7.04e-07), MRI abnormalities (HR = 1.59, p = 4.1e-04), cytogenetic abnormalities (HR = 1.86, p = 1.15e-06), and high MRPL13 expression (HR = 1.74, p = 1.25e-05) were all significantly correlated with shorter EFS.

Multivariate Cox regression confirmed that MRPL13 remained an independent prognostic factor, even after adjustment for established clinical covariates, for both OS (HR = 1.54, 95% CI: 1.11–2.13, p = 0.009) and EFS (HR = 1.43, 95% CI: 1.09–1.87, p = 0.0099). Other independent predictors included low albumin (OS: HR = 0.53, p = 4.0e-04; EFS: HR = 0.66, p = 0.003), high LDH (OS: HR = 1.81, p = 8.0e-04; EFS: HR = 1.54, p = 0.0024), MRI abnormalities (OS: HR = 1.55, p = 0.0111; EFS: HR = 1.37, p = 0.0281), and cytogenetic abnormalities (OS: HR = 1.88, p = 2.0e-04; EFS: HR = 1.61, p = 5.0e-04) (**Table 2**).

**Table 2:** Univariate and multivariate analysis for OS and EFS in GSE24080 MM patients.

Variables	OS				EFS			
	Univariate analysis		Multivariate analysis		Univariate analysis		Multivariate analysis	
	HR (95% CI)	*P* value	HR (95% CI)	*P* value	HR (95% CI)	*P* value	HR (95% CI)	*P* value
Age (<60 vs >=60)	1.42 (1.05–1.91)	0.0229			1.21 (0.939–1.55)	0.141		
ALB (low vs high)	0.482 (0.356–0.653)	0.00000246	0.53 (0.38–0.75)	0.0004	0.599 (0.467–0.769)	0.0000561	0.66 (0.5–0.87)	0.003
ASPC (low vs high)	1.88 (1.36–2.59)	0.000125	1.59 (1.03–2.47)	0.0376	1.6 (1.24–2.08)	0.000384	1.35 (0.95–1.92)	0.0992
B2M (low vs high)	2.3 (1.68–3.15)	0.000000238	1.34 (0.82–2.2)	0.2407	2.01 (1.55–2.59)	9.67E-08	1.46 (0.98–2.19)	0.0651
BMPC (low vs high)	1.53 (1.13–2.09)	0.00676	0.9 (0.59–1.37)	0.6106	1.47 (1.14–1.9)	0.00323	0.93 (0.66–1.33)	0.7054
CREAT (low vs high)	1.26 (0.927–1.71)	0.14			1.23 (0.957–1.59)	0.105		
CRP (low vs high)	1.51 (1.12–2.05)	0.00771	1.02 (0.73–1.43)	0.8912	1.27 (0.988–1.63)	0.0624		
Cyto.Abn (No vs Yes)	2.29 (1.7–3.1)	6.01E–08	1.88 (1.35–2.62)	0.0002	1.86 (1.45–2.38)	0.00000115	1.61 (1.23–2.12)	0.0005
FISH_1q21_Amplification (na vs 2 vs 3 vs 4+)	1.05 (0.931–1.17)	0.456			0.936 (0.853–1.03)	0.164		
GENDER (Female vs male)	0.968 (0.714–1.31)	0.836			0.99 (0.768–1.28)	0.936		
HGB (low vs high)	0.662 (0.489–0.896)	0.00756	1.13 (0.78–1.64)	0.5206	0.689 (0.536–0.884)	0.00345	1 (0.73–1.36)	0.9963
ISOTYPE (FLC vs IgA vs IgD vs IgG vs non-secretory)	0.98 (0.866–1.11)	0.746			0.992 (0.896–1.1)	0.873		
ISS (I vs II vs III)	1.7 (1.43–2.03)	3.38E–09	1.19 (0.88–1.6)	0.2539	1.54 (1.33–1.79)	1.05E-08	1.07 (0.83–1.38)	0.6231
LDH (low vs high)	2.19 (1.6–3)	0.00000101	1.81 (1.28–2.57)	0.0008	1.79 (1.39–2.31)	0.00000704	1.54 (1.17–2.04)	0.0024
MRI (low vs high)	1.86 (1.36–2.55)	0.00011	1.55 (1.11–2.18)	0.0111	1.59 (1.23–2.06)	0.00041	1.37 (1.03–1.8)	0.0281
RACE (White vs Other)	0.944 (0.586–1.52)	0.813			0.678 (0.438–1.05)	0.0828		
Subgroup (CD1 vs CD2 vs HY vs LB vs MF vs MS vs MY vs PR)	1.08 (1–1.16)	0.0414			1.05 (0.994–1.12)	0.0762		
MRPL13 (low vs high)	2.01 (1.49–2.71)	0.00000541	1.54 (1.11–2.13)	0.009	1.74 (1.36–2.23)	0.0000125	1.43 (1.09–1.87)	0.0099

External validation using the GSE136337 cohort (n = 426) further supported these findings. Patients in the high-expression group (n = 338) had significantly shorter median OS compared with the low-expression group (n = 88) (81.86 ± 44.60 months vs. 105.23 ± 45.08 months, p < 0.0001). Moreover, the high-expression group showed higher proportions of ISS-R stage III (17.96% vs. 7.06%, p = 0.0015), female patients (42.01% vs. 26.14%, p = 0.0093), low albumin (51.04% vs. 30.68%, p = 0.001), and high LDH (53.73% vs. 36.78%, p = 0.007) (**Supplementary Table 3**).

In Cox regression analysis of the GSE136337 cohort, high MRPL13 expression was significantly associated with both OS (HR = 1.68, 95% CI: 1.14–2.49, p = 0.00863) and PFS (HR = 1.95, 95% CI: 1.36-2.78, p = 2.57e-04) in univariate models. After multivariate adjustment, MRPL13 expression remained an independent predictor of poor outcomes: OS (HR = 1.69, 95% CI: 1.13–2.53, p = 0.0112) and PFS (HR = 1.86, 95% CI: 1.28-2.70, p = 0.00105), alongside ISS stage and molecular subtype (**Supplementary Table 4**).

Collectively, these results demonstrate that MRPL13 overexpression consistently predicts inferior survival outcomes across multiple independent cohorts, independent of other clinical and molecular risk factors, highlighting its potential utility as a prognostic biomarker for patient risk stratification in MM.

To further explore its potential predictive value, we conducted an exploratory analysis in the GSE9782 cohort, which included patients treated with bortezomib-based therapy. Patients were stratified into MRPL13-high and MRPL13-low expression groups, and treatment response (responders vs non-responders) was compared between the two groups. The response rate was 49.4% in the MRPL13-low group and 42.9% in the MRPL13-high group; however, no statistically significant difference was observed (Fisher’s exact test, P = 0.406). The corresponding odds ratio was 0.77 (95% CI: 0.43–1.37), indicating no clear association between MRPL13 expression and treatment response in this cohort.

### 3.5 Expression patterns and functional heterogeneity of MRPL13 within MM plasma cell/tumor subclusters

**Figure 4:**
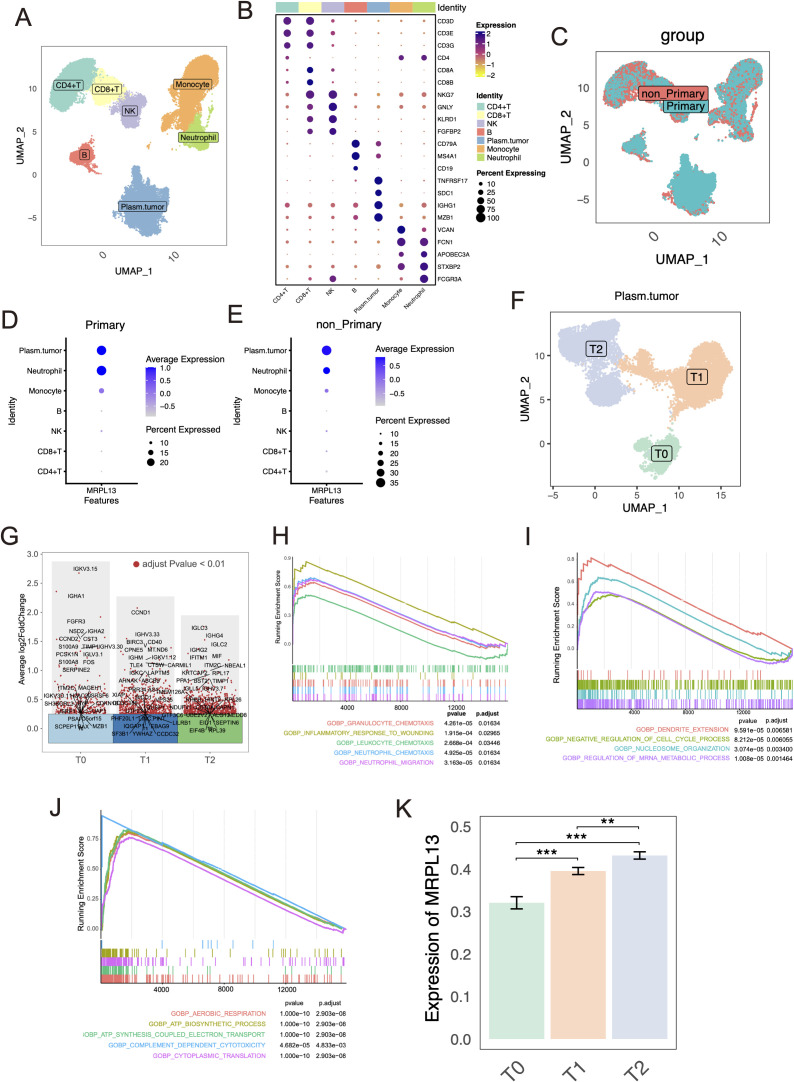
Single-cell transcriptomic landscape of MM patients. **(A)** UMAP clustering of 74,986 cells from 24 MM patients, identifying seven major cell populations; **(B)** Bubble plot of canonical marker genes across cell types; **(C)** UMAP projection of primary versus relapsed/non-primary MM patients; **(D, E)** Bubble plots of MRPL13 expression in different cell populations from primary **(D)** and non-primary **(E)** patients; **(F)** Subclustering of Plasma/Tumor cells into three subpopulations (T0, T1, and T2); **(G)** Volcano plots of differentially expressed genes among T0, T1, and T2; **(H–J)** GSEA plots showing functional enrichment of subgroup-specific genes; **(K)** Comparative expression of MRPL13 across T0, T1, and T2 subpopulations.

We integrated single-cell transcriptomic data from 74,986 cells derived from 24 MM patient samples to characterize the cellular landscape. Seven major populations were identified, including CD4^+^ T cells, CD8^+^ T cells, natural killer (NK) cells, B cells, plasma/tumor cells (Plasma/Tumor), monocytes, and neutrophils. Each lineage was annotated based on canonical marker expression, confirming accurate cluster assignment: CD4^+^ T cells (CD3D, CD3E, CD3G, and CD4) (18); CD8^+^ T cells (CD3D, CD3E, CD3G, CD8A, and CD8B) (19); NK cells (NKG7, GNLY, KLRD1, and FGFBP2) (20); B cells (CD79A, MS4A1, and CD19) (21); plasma/tumor cells (TNFRSF17/BCMA, SDC1/CD138, IGHG1, and MZB1) (22); monocytes (VCAN, FCN1, and APOBEC3A); and neutrophils (STXBP2 and FCGR3A) (**Figures 4A, B**; **Supplementary Tables 5**, **6**). In addition, the UMAP projection revealed a clear separation between primary and non-primary MM samples (**Figure 4C**), indicating distinct transcriptomic profiles across sample sources. Notably, MRPL13 was most highly expressed in Plasma/Tumor cells, with moderate expression in monocytes and neutrophils (**Figures 4D, E**).

Further reclustering of Plasma/Tumor cells revealed three transcriptionally distinct subclusters (T0, T1, and T2), each displaying unique molecular signatures and functional pathways. T0 cells exhibited high expression of FGFR3, NSD2, CCND2, S100A8, S100A9, FOS, SERPINE2, and CST3 and were enriched for neutrophil chemotaxis, inflammatory responses, and immune cell recruitment pathways. This profile mirrors the molecular hallmark of t(4;14)-positive MM, characterized by FGFR3/NSD2 co-activation and CCND2 upregulation, forming a canonical oncogenic triad that drives proliferation and microenvironmental remodeling. T1 cells were characterized by CCND1, IGHV3.33, CD40, PRKCB, IGHM, CPNE5, TLE4, and CARMIL1 expression and were enriched for negative regulation of the cell cycle, mRNA metabolic processes, and nucleosome organization. The defining feature was CCND1 overexpression, consistent with t(11;14)-associated MM, representing a phenotype of poorly-proliferative but apoptosis-resistant malignant plasma cells. T2 cells were characterized by IGHG2, IGHG4, RPL17, LDHA, MIF, BST2, IFITM1, IGLC2, and IGLC3, with enrichment for cytoplasmic translation, ATP biosynthesis, aerobic respiration, and complement-mediated cytotoxicity. This subcluster displayed a hypermetabolic and stress-adaptive phenotype, suggesting a potential role in disease progression and therapy resistance (**Figures 4F–J**). Consistently, Gene Ontology (GO) Biological Process analysis further demonstrated that MRPL13-high cells were significantly enriched for mitochondrial gene expression, mitochondrial translation, and mitochondrial RNA metabolic processes, suggesting a potential association with mitochondrial biosynthetic and metabolic activity (**Supplementary Figure 2**).

Importantly, MRPL13 expression differed significantly across the three Plasma/Tumor subclusters, with the highest levels observed in T2, followed by T1, and the lowest in T0 (p < 0.05 for all comparisons; **Figure 4K**). This gradient strongly suggests that MRPL13 overexpression is closely linked to the heightened translational and metabolic activity of the T2 subpopulation. Functional enrichment aligned with MRPL13 biology, indicating that MRPL13 may serve as a key driver of protein synthesis and energy metabolism in aggressive MM subsets, thereby contributing to disease progression.

### 3.6 Single-cell analysis reveals that high MRPL13 expression is closely associated with immune suppression and tumor immune evasion

**Figure 5:**
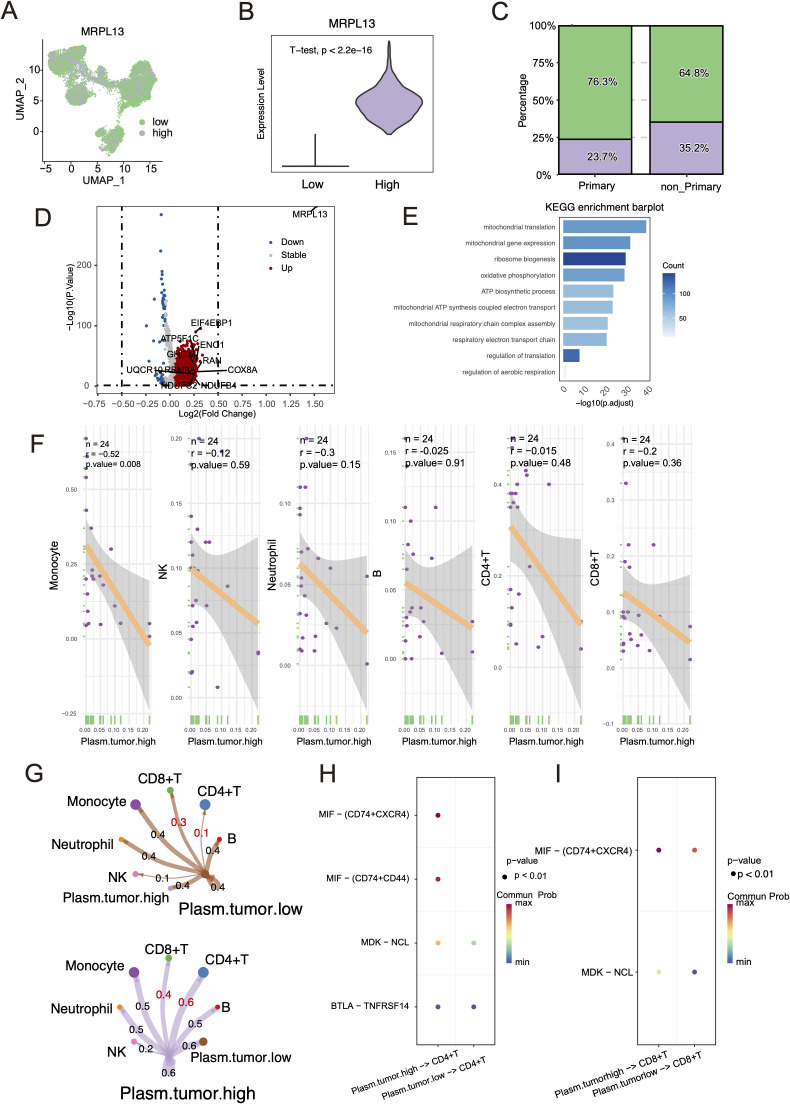
Functional analysis of MRPL13 and its association with the immune microenvironment. **(A, B)** UMAP and violin plots depicting MRPL13 expression distribution; **(C)** Proportion of MRPL13-high versus MRPL13-low Plasma/Tumor cells in primary and non-primary patients; **(D)** Volcano plot of MRPL13-associated differentially expressed genes; **(E)** KEGG pathway enrichment of MRPL13-related genes; **(F)** Correlation between MRPL13 expression and immune cell infiltration; **(G)** Cell–cell interaction network in MRPL13-high versus MRPL13-low groups; **(H, I)** Upregulated ligand–receptor interactions in MRPL13-high MM cells.

To investigate the link between MRPL13 and immune suppression, we stratified Plasma/Tumor cells into high- and low-expression groups based on MRPL13 levels (**Figures 5A, B**). Notably, the proportion of MRPL13-high tumor cells was significantly greater in non-primary patients compared with newly diagnosed cases (35.2% vs. 23.7%; **Figure 5C**), suggesting that MRPL13-high cells are enriched in relapsed or advanced disease.

Differential expression analysis revealed significant upregulation of EIF4EBP1, ATP5F1C, END1, and GITH1 in the MRPL13-high group, with functional enrichment of mitochondrial translation, mitochondrial gene expression, ribosome biogenesis, and oxidative phosphorylation (**Figures 5D, E**). These findings indicate that MRPL13-high tumor cells exhibit a more active metabolic and protein synthesis program.

In the immune microenvironment analysis, the proportion of monocytes was significantly reduced with increasing abundance of MRPL13-high tumor cells (r = -0.52, p = 0.008), while other immune subsets, including NK cells, neutrophils, B cells, CD4^+^ T cells, and CD8^+^ T cells, also showed decreasing trends without reaching statistical significance (**Figure 5F**). This suggests that expansion of MRPL13-high tumor cells is associated with diminished monocyte infiltration and may concurrently suppress other immune compartments.

Cell–cell communication analysis further demonstrated that MRPL13-high tumor cells exhibited markedly enhanced interactions with T cells (**Figure 5G**). At the ligand–receptor level, their interactions with CD4^+^ T cells were mediated predominantly through the MIF-(CD74+CXCR4), MIF-(CD74+CD44), MDK–NCL, and BTLA–TNFRSF14 axes, while interactions with CD8^+^ T cells were strengthened mainly via the MIF-(CD74+CXCR4) and MDK–NCL pathways (**Figures 5H, I**).

Collectively, these results suggest that MRPL13-high tumor cells are not only linked to reduced immune infiltration but also reinforce suppressive ligand–receptor interactions with CD4^+^ and CD8^+^ T cells, thereby fostering an immune-evasive tumor microenvironment.

### 3.7 Downregulation of MRPL13 significantly suppresses MM cell proliferation and induces G0/G1-phase arrest

**Figure 6:**
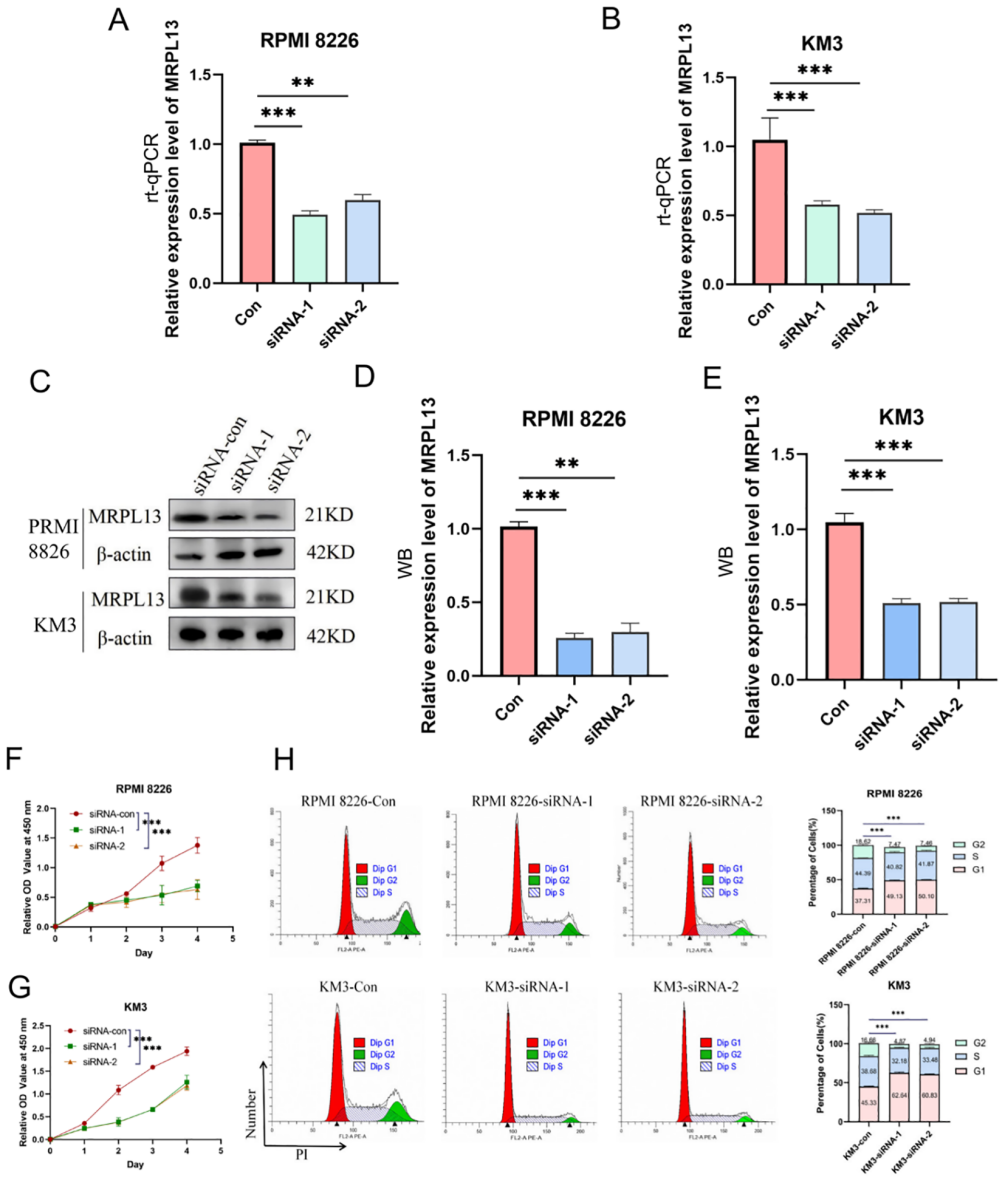
Functional validation of MRPL13 knockdown *in vitro*. **(A)** RT-qPCR analysis confirming MRPL13 knockdown efficiency in RPMI-8226 cells following siRNA transfection. **(B)** RT-qPCR analysis confirming MRPL13 knockdown efficiency in KM3 cells following siRNA transfection. **(C–E)** Western blot analysis confirming effective silencing of MRPL13 protein expression in RPMI-8226 and KM3 cells. **(F, G)** CCK-8 assays demonstrating reduced proliferation of MM cells upon MRPL13 knockdown in RPMI-8226 and KM3 cells. **(H)** Flow cytometric analysis showing G0/G1 cell-cycle arrest induced by MRPL13 silencing. Statistical significance is indicated as ns (not significant), *p < 0.05, **p < 0.01, ***p < 0.001.

Building upon the finding that high MRPL13 expression predicts poor prognosis in MM, we further investigated its potential as a therapeutic target through *in vitro* functional assays. In the RPMI 8226 and KM3 cell lines, MRPL13 expression was silenced using two distinct small interfering RNAs (siRNA-1 and siRNA-2). RT-qPCR analysis demonstrated that both siRNAs markedly reduced MRPL13 mRNA levels compared with controls (RPMI 8226: p < 0.01 and p < 0.001; KM3: both p < 0.001) (**Figures 6A, B**). Consistently, Western blot analysis confirmed significant suppression of MRPL13 protein expression in both cell lines (**Figures 6C–E**).

Functionally, CCK-8 proliferation assays revealed that MRPL13 knockdown significantly impaired the proliferative capacity of RPMI 8226 and KM3 cells, with the inhibitory effect most pronounced at 3–4 days post-transfection (p < 0.001) (**Figures 5F, G**). Moreover, flow cytometric analysis of the cell cycle indicated that MRPL13 silencing substantially increased the proportion of cells in the G1 phase, accompanied by a concomitant decrease in S-phase cells, indicative of G1-phase arrest (p < 0.001) (**Figure 6H**).

Collectively, these results suggest that MRPL13 exerts a pro-proliferative role in MM cells, and its downregulation effectively suppresses tumor cell growth by inducing G0/G1-phase arrest, thereby supporting MRPL13 as a potential molecular target for MM therapy.

## 4 Discussion

Multiple myeloma (MM) is a hematologic malignancy characterized by the clonal proliferation of bone marrow plasma cells, ranking as the second most common blood cancer after non-Hodgkin lymphoma (23). In 2022, an estimated 188,000 new MM cases and 121,000 deaths occurred worldwide, with an age-standardized incidence rate of 1.9 per 100,000 and a persistently rising trend (24). Clinically, MM is typified by end-organ damage, including renal impairment, hypercalcemia, osteolytic lesions, and anemia. Despite remarkable therapeutic advances with proteasome inhibitors, immunotherapy, and stem cell transplantation (25, 26), overall survival has significantly improved. Nevertheless, MM remains a heterogeneous disease, and the majority of patients ultimately relapse or develop drug resistance. Consequently, the identification of novel diagnostic and prognostic biomarkers, in addition to actionable therapeutic targets, remains a pressing need.

In recent years, mitochondrial dysfunction has emerged as a critical hallmark in cancer biology (27). Mitochondrial ribosomal proteins (MRPs), as essential components of mitochondrial protein synthesis, play pivotal roles in sustaining mitochondrial translational activity and cellular energy metabolism, thereby supporting tumor cell growth and survival (6). Previous studies have reported that MRPs may be associated with oncogenic signaling pathways, such as PI3K/AKT/mTOR and MYC targets, in various malignancies, including breast, lung, and colorectal cancers (28). Among them, mitochondrial ribosomal protein L13 (MRPL13) has been reported to be dysregulated in several cancers and may be involved in tumor progression through the regulation of mitochondrial protein synthesis and metabolic activity (29).

However, the biological significance and clinical relevance of MRPL13 in MM have not been systematically explored. By integrating clinical and molecular data from 3,117 MM patients across GEO and TCGA_MMRF datasets, our study comprehensively evaluated the prognostic value of MRPL13. We found that elevated MRPL13 expression was consistently associated with inferior OS, event-free survival (EFS), and progression-free survival (PFS) and was validated as an independent adverse prognostic factor across multiple independent cohorts. Furthermore, single-cell transcriptomic analysis and *in vitro* functional experiments provided mechanistic insights, demonstrating that MRPL13 may drive MM progression through metabolic reprogramming, dysregulation of protein translation, and modulation of the immune microenvironment. Collectively, our findings establish MRPL13 as a robust prognostic biomarker and a promising therapeutic target in MM.

First, the consistent findings across large, multicenter cohorts substantially strengthen the robustness of our conclusions. Notably, in independent datasets, including GSE24080, GSE2658, GSE136337, and TCGA_MMRF, patients with high MRPL13 expression consistently exhibited significantly shorter OS and PFS, suggesting that MRPL13 may serve as a broadly applicable prognostic marker across diverse patient populations. Importantly, in multivariable Cox regression models adjusting for critical clinical parameters, such as ISS stage and molecular subtype, MRPL13 retained its prognostic significance, underscoring its potential utility in clinical risk stratification.

High MRPL13 expression was closely associated with adverse clinical features in MM, including ISS stage III, 1q21 amplification, cytogenetic abnormalities, elevated LDH, and hypoalbuminemia—all of which are well-established indicators of poor prognosis. Particularly, MRPL13 expression was markedly enriched in the proliferation (PR) molecular subtype, which is typically characterized by higher oncogenic potential and inferior clinical outcomes, suggesting that MRPL13 may play a critical role in the pathogenesis of this aggressive MM subset. Mechanistically, MRPL13 was significantly upregulated in proliferative MM and enriched in pathways strongly linked to tumor progression, such as PI3K/AKT/mTOR, MYC target activation, and G2/M checkpoint regulation (29). Given that MYC translocations occur in approximately 30%–50% of newly diagnosed MM (NDMM) and are strongly associated with high tumor burden and adverse prognosis (30), these findings collectively support the hypothesis that MRPL13 may act as an important driver of MM progression.

Single-cell transcriptomic analysis further refined our understanding of MRPL13 biology. We identified three functionally heterogeneous malignant plasma cell subpopulations (T0/T1/T2), among which the T2 subset exhibited a highly active metabolic phenotype with pronounced MRPL13 expression. This suggests that MRPL13 may serve as a critical regulator in sustaining the elevated translational and metabolic activity characteristic of this subpopulation. Functional enrichment analyses corroborated these observations, showing that MRPL13-high cells significantly upregulated oxidative phosphorylation, mitochondrial protein synthesis, and ribosomal pathways, in line with its role as a structural component of the mitochondrial large ribosomal subunit.

Of particular interest, high MRPL13 expression was associated with alterations in the immune microenvironment. As the proportion of MRPL13-high cells increased, bone marrow samples from MM patients showed a decrease in T cells, NK cells, and monocytes, although only the reduction in monocytes reached statistical significance, accompanied by changes in immune-related ligand–receptor pairs such as MIF–CD74 and MDK–NCL. These findings suggest that MRPL13 may be associated with tumor immune escape and disease progression through alterations in immune cell composition (29). In addition, functional enrichment analysis derived from single-cell transcriptomic data provided further mechanistic insight. MRPL13-high cells were significantly enriched in mitochondrial gene expression, mitochondrial translation, and ribosomal pathways, indicating enhanced mitochondrial biosynthetic and metabolic activity in these cells. Given the central role of mitochondrial function in maintaining cellular energy homeostasis, these metabolic alterations may contribute to cellular state reprogramming and provide a potential mechanistic explanation for the impaired proliferative capacity and G1-phase cell cycle arrest observed upon MRPL13 knockdown. This observation is consistent with the clinical enrichment of MRPL13-high cells in relapsed or advanced-stage patients, further supporting its potential role in shaping the multiple myeloma immune microenvironment. However, it should be noted that MRPL13 expression in this study was primarily evaluated at the transcriptomic level, and the observed relationships are based on correlation analyses without functional validation of immune regulatory mechanisms.

Functional studies further corroborated the pro-tumorigenic role of MRPL13. Knockdown of MRPL13 in RPMI-8226 and KM3 cells significantly impaired cell proliferation and induced G1-phase cell cycle arrest, underscoring its role in sustaining MM cell growth. Collectively, these findings reinforce the clinical correlations at a functional level and support MRPL13 as a potential therapeutic target in multiple myeloma. However, MRPL13 expression in this study was primarily evaluated at the transcriptomic level. Although protein expression was validated in MM cell lines by Western blot analysis, protein-level validation in primary patient-derived samples was not performed. This limitation should be considered when interpreting the translational relevance of the present findings.

Nevertheless, several limitations should be acknowledged. Although our conclusions were validated across multiple large-scale datasets, inherent heterogeneity in the GEO and TCGA_MMRF cohorts and incomplete clinical annotations warrant cautious interpretation and prospective clinical validation. In addition, while single-cell data provided preliminary insight into the association between MRPL13 and the immune microenvironment, the precise regulatory mechanisms remain undefined and will require in-depth mechanistic studies, including studies using *in vivo* models. Finally, as no pharmacological inhibitors of MRPL13 currently exist, its translational potential as a therapeutic target will depend on advances in drug development and preclinical validation. These findings suggest that MRPL13 represents a stable prognostic biomarker in multiple myeloma; however, its potential role as a predictive biomarker for treatment response requires further validation in larger cohorts with more comprehensive treatment annotation. In addition, although MRPL13 knockdown significantly inhibited cell proliferation and induced G0/G1 arrest, direct drug sensitivity assays following gene silencing were not performed, which limits the assessment of its role in modulating treatment response. Further studies incorporating functional drug-response assays and larger treatment-annotated cohorts are warranted to clarify its predictive and therapeutic relevance.

## 5 Conclusion

This study represents the first comprehensive evaluation of MRPL13 expression and its clinical significance in multiple myeloma. We demonstrated that elevated MRPL13 expression is strongly associated with adverse prognosis and retains independent predictive value beyond established clinical and molecular risk factors. Mechanistically, MRPL13 may contribute to MM progression through the regulation of cellular metabolism, oncogenic signaling pathways, and remodeling of the immune microenvironment. By integrating large-scale transcriptomic analyses, single-cell profiling, and functional validation, our findings establish MRPL13 as a promising prognostic biomarker and potential therapeutic target in MM with significant translational potential. Prospective studies and clinical validation are warranted to further elucidate its role and advance its application in precision medicine for MM.

## Data Availability

The original contributions presented in the study are included in the article/**Supplementary Material**. Further inquiries can be directed to the corresponding authors.

## Supplementary material

Supplementary Table 6 All Supplementary Table can be found and accessed at the following URL: https://figshare.com/articles/dataset/Supplementary_Table_of_the_article_MRPL13_represents_an_independent_prognostic_biomarker_and_a_potential_therapeutic_target_in_multiple_myeloma/30416119.

## References

[1] Rajkumar SV . Multiple myeloma: 2024 update on diagnosis, risk-stratification, and management. Am J Hematol. (2024) 99:1802–24. doi: 10.1002/ajh.27422 38943315 PMC11404783

[2] van de Donk N Pawlyn C Yong KL . Multiple myeloma. Lancet (London England). (2021) 397:410–27. doi: 10.1016/s0140-6736(21)00135-5 33516340

[3] Xu L Wen C Xia J Zhang H Liang Y Xu X . Targeted immunotherapy: harnessing the immune system to battle multiple myeloma. Cell Death Discov. (2024) 10:55. doi: 10.1038/s41420-024-01818-6 38280847 PMC10821908

[4] Rodriguez-Otero P Usmani S Cohen AD van de Donk N Leleu X Gállego Pérez-Larraya J . International Myeloma Working Group immunotherapy committee consensus guidelines and recommendations for optimal use of T-cell-engaging bispecific antibodies in multiple myeloma. Lancet Oncol. (2024) 25:e205–16. doi: 10.1016/s1470-2045(24)00043-3 38697166

[5] Du H Xu T Yu S Wu S Zhang J . Mitochondrial metabolism and cancer therapeutic innovation. Signal Transduction Targeted Ther. (2025) 10:245. doi: 10.1038/s41392-025-02311-x 40754534 PMC12319113

[6] Huang G Li H Zhang H . Abnormal expression of mitochondrial ribosomal proteins and their encoding genes with cell apoptosis and diseases. Int J Mol Sci. (2020) 21. doi: 10.3390/ijms21228879 33238645 PMC7700125

[7] Jing C Fu R Wang C Li X Zhang W . MRPL13 act as a novel therapeutic target and could promote cell proliferation in non-small cell lung cancer. Cancer Manage Res. (2021) 13:5535–45. doi: 10.2147/cmar.s316428 34285575 PMC8285246

[8] Lee YK Lim JJ Jeoun UW Min S Lee EB Kwon SM . Lactate-mediated mitoribosomal defects impair mitochondrial oxidative phosphorylation and promote hepatoma cell invasiveness. J Biol Chem. (2017) 292:20208–17. doi: 10.1074/jbc.m117.809012 28978646 PMC5724007

[9] Foundation MMR . MMRF Commpass Study (MMRF-COMMPASS, NCT01454297). Norwalk, CT: Multiple Myeloma Research Foundation (2011).

[10] Shi L Campbell G Jones WD Campagne F Wen Z Walker SJ . The MicroArray Quality Control (MAQC)-II study of common practices for the development and validation of microarray-based predictive models. Nat Biotechnol. (2010) 28:827–38. doi: 10.1038/nbt.1665 PMC3315840 20676074

[11] Zhan F Huang Y Colla S Stewart JP Hanamura I Gupta S . The molecular classification of multiple myeloma. Blood. (2006) 108:2020–8. doi: 10.1182/blood-2005-11-013458 16728703 PMC1895543

[12] Mulligan G Mitsiades C Bryant B Zhan F Chng WJ Roels S . Gene expression profiling and correlation with outcome in clinical trials of the proteasome inhibitor bortezomib. Blood. (2007) 109:3177–88. doi: 10.1182/blood-2006-09-044974 17185464

[13] Heuck CJ Qu P van Rhee F Waheed S Usmani SZ Epstein J . Five gene probes carry most of the discriminatory power of the 70-gene risk model in multiple myeloma. Leukemia. (2014) 28:2410–3. doi: 10.1182/blood.v122.21.125.125 PMC4274609 25079174

[14] Shaughnessy JD Jr. Zhan F Burington BE Huang Y Colla S Hanamura I . A validated gene expression model of high-risk multiple myeloma is defined by deregulated expression of genes mapping to chromosome 1. Blood. (2007) 109:2276–84. doi: 10.1182/blood-2006-07-038430 17105813

[15] Danziger SA McConnell M Gockley J Young MH Rosenthal A Schmitz F . Bone marrow microenvironments that contribute to patient outcomes in newly diagnosed multiple myeloma: a cohort study of patients in the Total Therapy clinical trials. PloS Med. (2020) 17:e1003323. doi: 10.1371/journal.pmed.1003323 33147277 PMC7641353

[16] Livak KJ Schmittgen TD . Analysis of relative gene expression data using real-time quantitative PCR and the 2(-Delta Delta C(T)) method. Methods (San Diego Calif). (2001) 25:402–8. doi: 10.1006/meth.2001.1262 11846609

[17] Towbin H Staehelin T Gordon J . Electrophoretic transfer of proteins from polyacrylamide gels to nitrocellulose sheets: procedure and some applications. PNAS. (1979) 76:4350–4. doi: 10.1073/pnas.76.9.4350 388439 PMC411572

[18] Liang X Lu W Wang LJB . Single-cell RNA sequencing of CAR-T cell infusion products reveals ‘viral mimicry’ as potential resistance mechanism. (2024) 144:7170. doi: 10.1182/blood-2024-198300

[19] Cheng D Zhang Z Liu D Mi Z Tao W Fu J . Unraveling T cell exhaustion in the immune microenvironment of osteosarcoma via single-cell RNA transcriptome. Cancer Immunology Immunotherapy CII. (2024) 73:35. doi: 10.1007/s00262-023-03585-2 38280005 PMC10821851

[20] Korst C Tahri S Duetz C Bruins WSC de Jonge AV de Jong ME . The bone marrow NK-cell profile predicts MRD negativity in patients with multiple myeloma treated with daratumumab-based therapy. Blood. (2025) 145:3007–14. doi: 10.1182/blood.2024026455 40019438

[21] Lerman I Bawany F Whitt W Esaa F Yon J Babkowski N . Prominent B-cell signature differentiates discoid from subacute cutaneous lupus erythematosus. J Invest Dermatol. (2022) 142:2885–95.e2. doi: 10.1016/j.jid.2022.03.033 35594909

[22] Yao L Wang JT Jayasinghe RG O'Neal J Tsai CF Rettig MP . Single-cell discovery and multiomic characterization of therapeutic targets in multiple myeloma. Cancer Res. (2023) 83:1214–33. doi: 10.1158/0008-5472.can-22-1769 36779841 PMC10102848

[23] Bray F Ferlay J Soerjomataram I Siegel RL Torre LA Jemal A . Global cancer statistics 2018: GLOBOCAN estimates of incidence and mortality worldwide for 36 cancers in 185 countries. CA: A Cancer J For Clin. (2018) 68:394–424. doi: 10.3322/caac.21492 30207593

[24] Mafra A Laversanne M Marcos-Gragera R Chaves HVS McShane C Bray F . The global multiple myeloma incidence and mortality burden in 2022 and predictions for 2045. J Natl Cancer Institute. (2025) 117:907–14. doi: 10.1093/jnci/djae321 39658225

[25] Razzak M . Immunotherapy: Engineering a strategy for multiple myeloma. Nat Rev Cancer. (2015) 15:514. doi: 10.1038/nrc4006 26289313

[26] Moreau P San Miguel J Sonneveld P Mateos MV Zamagni E Avet-Loiseau H . Multiple myeloma: ESMO clinical practice guidelines for diagnosis, treatment and follow-up. Ann Oncol Off J Eur Soc For Med Oncol. (2017) 28:iv52–61. doi: 10.1093/annonc/mdt297 28453614

[27] Wang SF Tseng LM Lee HC . Role of mitochondrial alterations in human cancer progression and cancer immunity. J BioMed Sci. (2023) 30:61. doi: 10.1186/s12929-023-00956-w 37525297 PMC10392014

[28] Cai M Li H Chen R Zhou X . MRPL13 promotes tumor cell proliferation, migration and EMT process in breast cancer through the PI3K-AKT-mTOR pathway. Cancer Manage Res. (2021) 13:2009–24. doi: 10.2147/cmar.s296038 33658859 PMC7920513

[29] Zhong X He Z Fan Y Yin L Hong Z Tong Y . Multi-omics analysis of MRPL-13 as a tumor-promoting marker from pan-cancer to lung adenocarcinoma. Aging. (2023) 15:10640–80. doi: 10.18632/aging.205104 37827692 PMC10599762

[30] Boyle EM Ashby C Tytarenko RG Deshpande S Wang H Wang Y . BRAF and DIS3 mutations associate with adverse outcome in a long-term follow-up of patients with multiple myeloma. Clin Cancer Res Off J Am Assoc For Cancer Res. (2020) 26:2422–32. doi: 10.1158/1078-0432.ccr-19-1507 31988198

