# Supplementary material for: Prognostic and biological significance of MRPL13 in multiple myeloma: evidence from multi-cohort, single-cell analyses
Source: Front Oncol. 2026 Aug 5;16:1880529. doi: 10.3389/fonc.2026.1880529 (PMC13485542; doi:10.3389/fonc.2026.1880529)
Supplement: Supplementary file 1 [file DataSheet1.pdf]

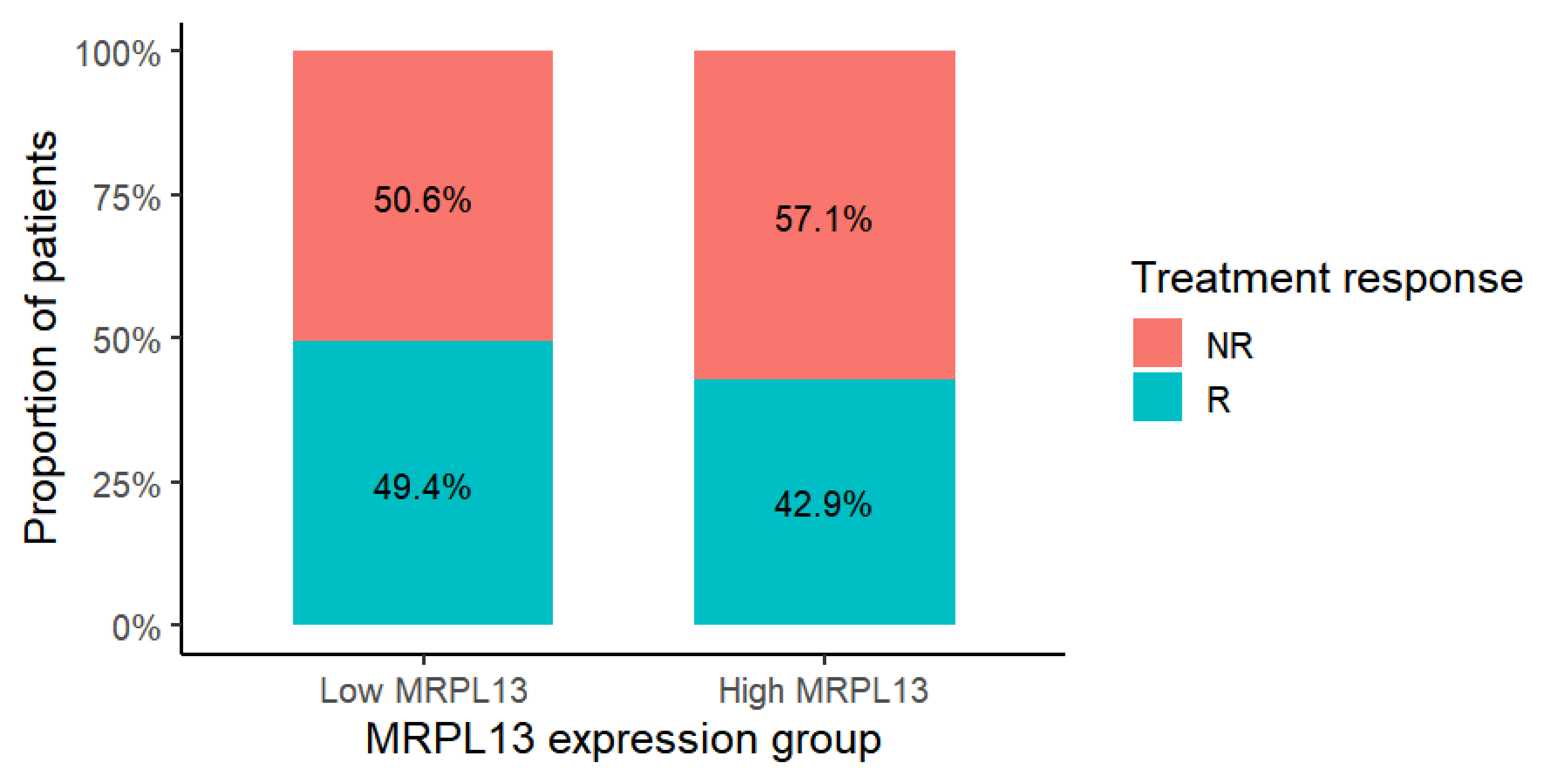
100%
75%
50%
25%
0%
Proportion of patients
50.6%
49.4%
57.1%
42.9%
Low MRPL13
High MRPL13
MRPL13 expression group
Treatment response
NR
R
