# Supplementary material for: Prognostic and biological significance of MRPL13 in multiple myeloma: evidence from multi-cohort, single-cell analyses
Source: Front Oncol. 2026 Aug 5;16:1880529. doi: 10.3389/fonc.2026.1880529 (PMC13485542; doi:10.3389/fonc.2026.1880529)
Supplement: Supplementary file 2 [file DataSheet2.pdf]

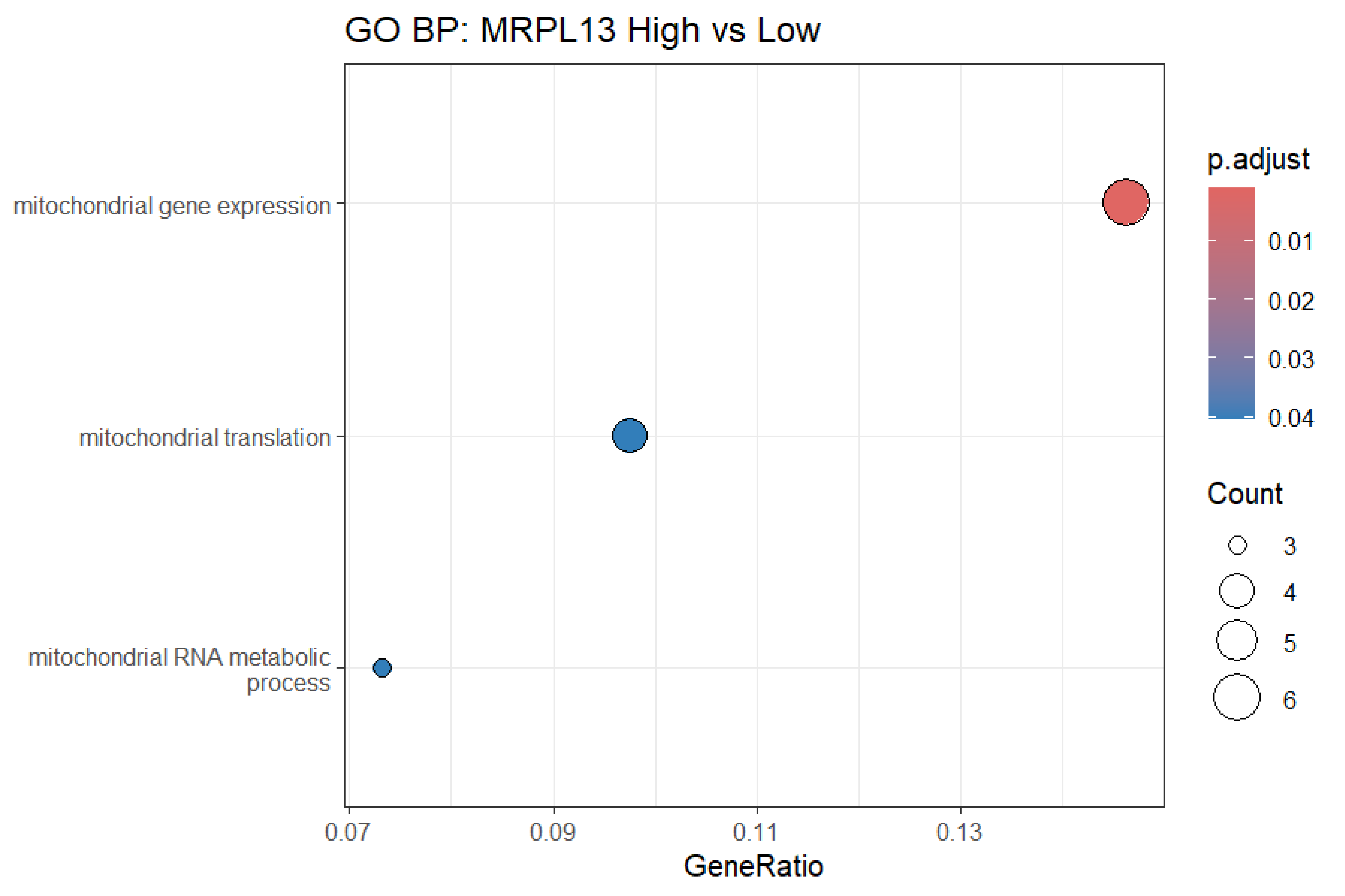
GO BP: MRPL13 High vs Low
mitochondrial gene expression
mitochondrial translation
mitochondrial RNA metabolic process
0.07
0.09
0.11
0.13
GeneRatio
p.adjust
0.01
0.02
0.03
0.04
Count
3
4
5
6
