# Supplementary material for: Prognostic and biological significance of MRPL13 in multiple myeloma: evidence from multi-cohort, single-cell analyses
Source: Front Oncol. 2026 Aug 5;16:1880529. doi: 10.3389/fonc.2026.1880529 (PMC13485542; doi:10.3389/fonc.2026.1880529)
Supplement: Supplementary file 3 [file DataSheet3.pdf]

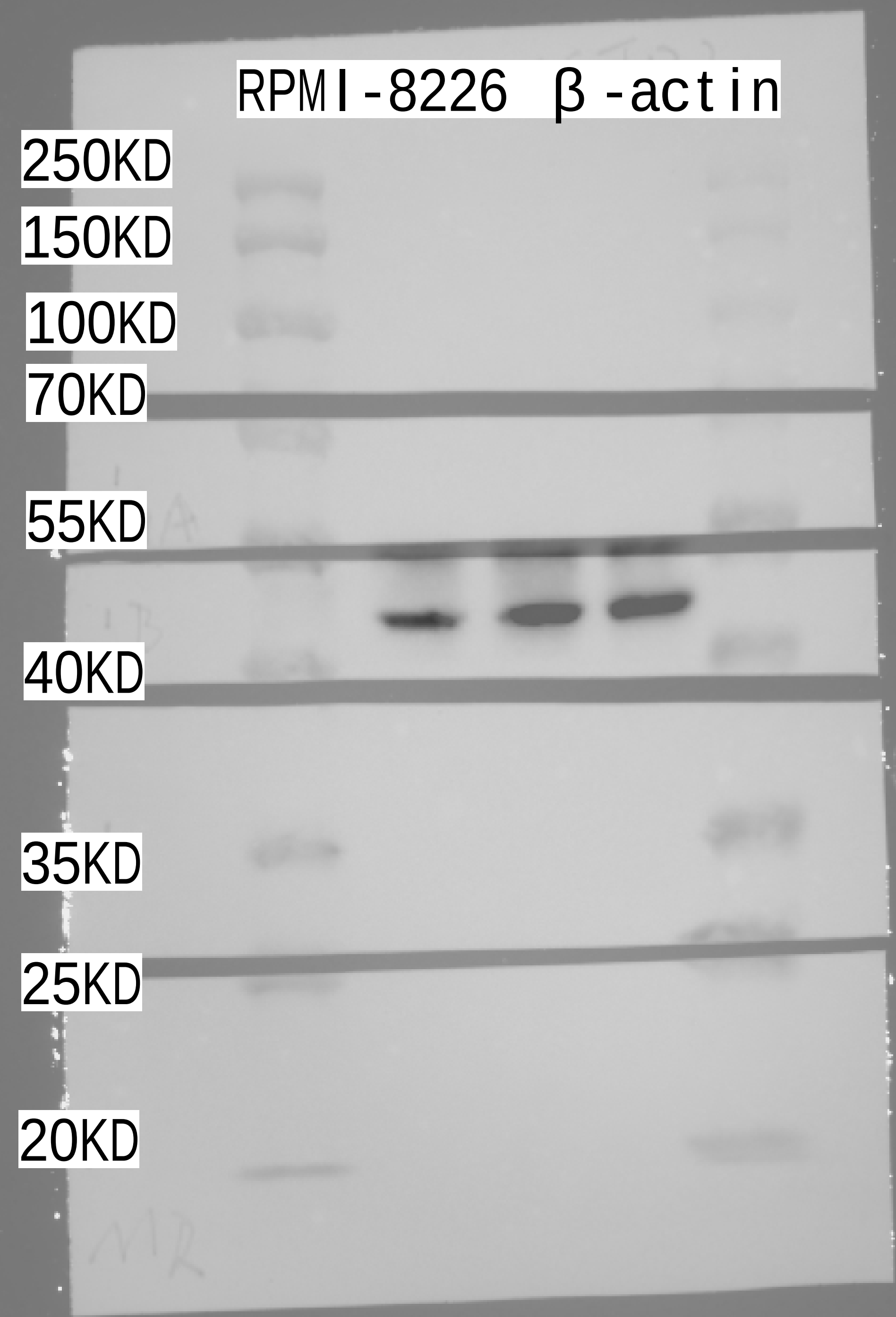
RPMI-8226 β-actin
250KD
150KD
100KD
70KD
55KD
40KD
35KD
25KD
20KD

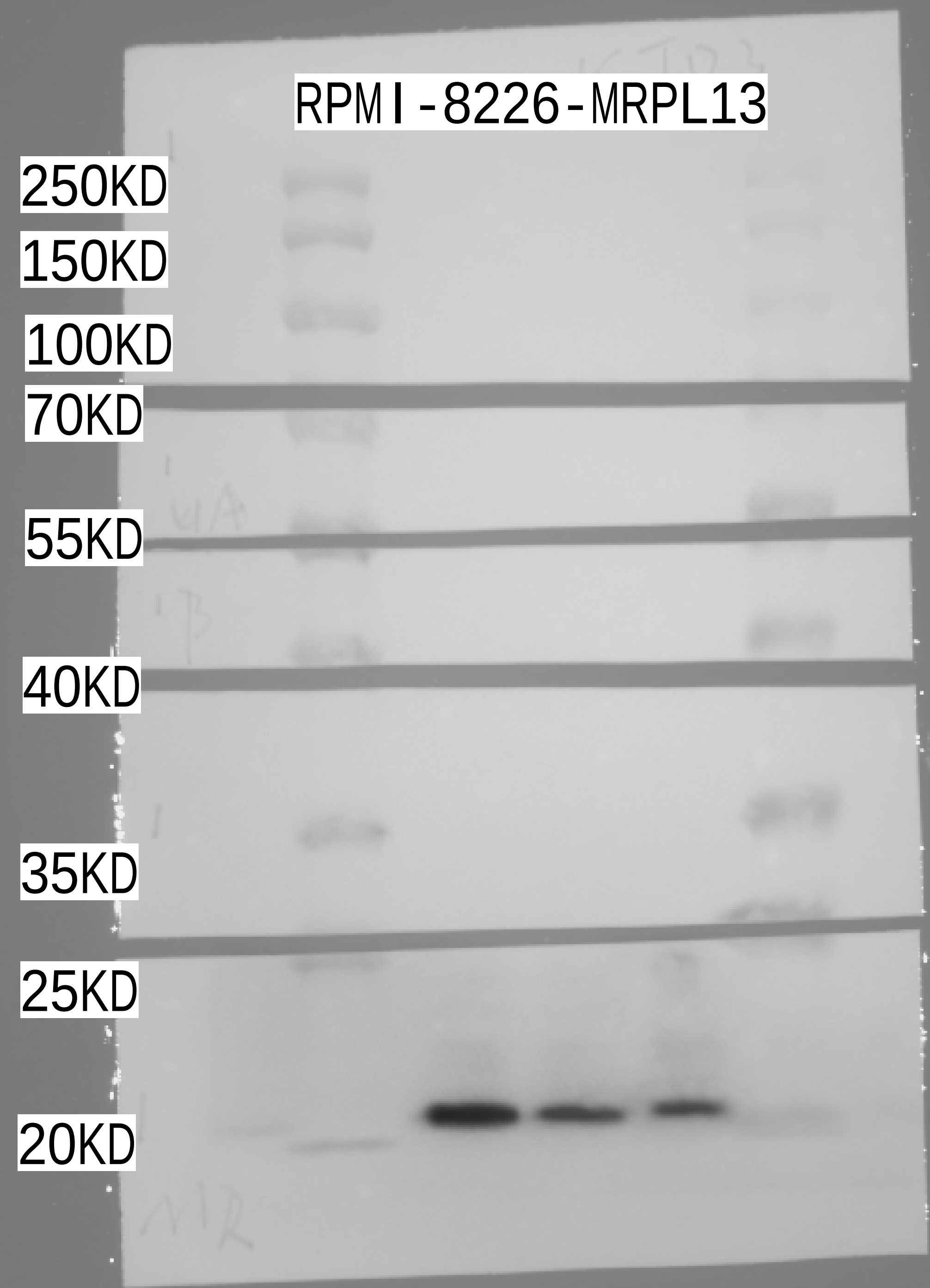
RPMI-8226-MRPL13
250KD
150KD
100KD
70KD
55KD
40KD
35KD
25KD
20KD

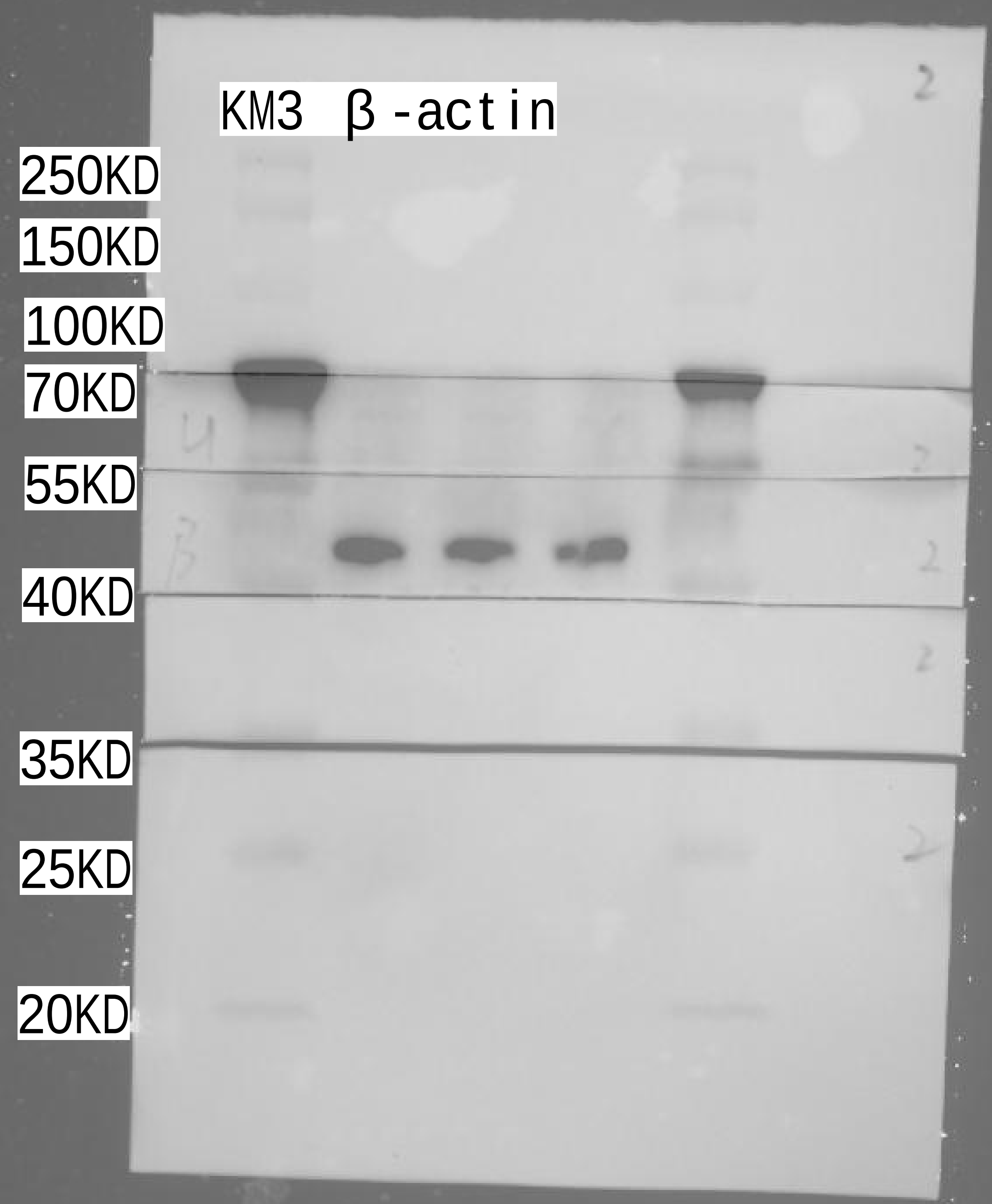
KM3 β-actin
250KD
150KD
100KD
70KD
55KD
40KD
35KD
25KD
20KD

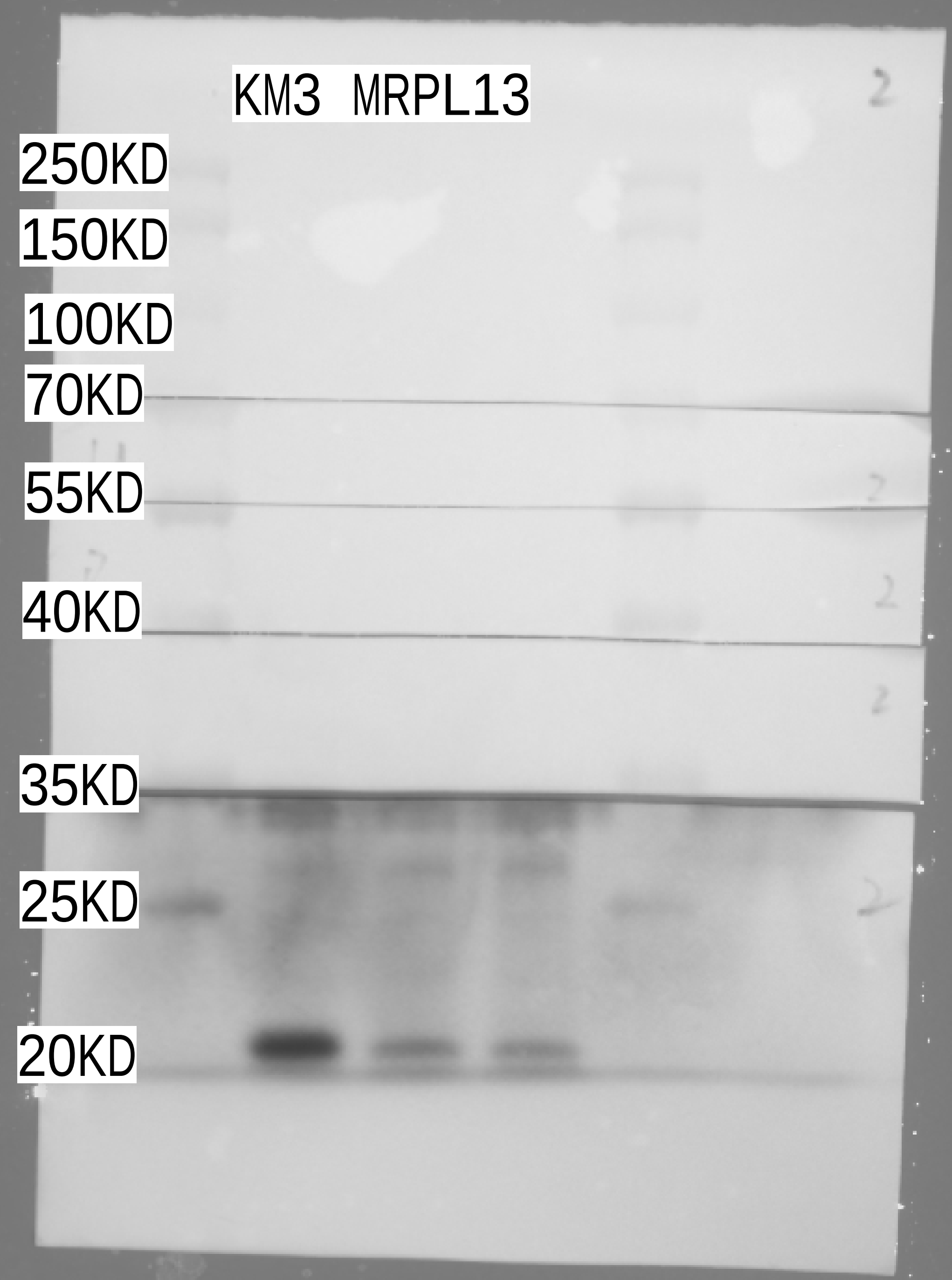
KM3 MRPL13
250KD
150KD
100KD
70KD
55KD
40KD
35KD
25KD
20KD
